# 596. Image Sensor-based Real Time Monitoring of Bacterial Growth on Agar Plates

**DOI:** 10.1093/ofid/ofad500.663

**Published:** 2023-11-27

**Authors:** Makoto Taketani, Tsubasa Inagaki, Mitsutaka Nakada, Reiichi Ariizumi, Masakazu Nakajima, Akihiko Fujisawa, Tomoya Tezen, Takanori Tsunashima, Kaoru Ito, Daichi Abe, Kazunori Yamaguchi, Kenichiro Ohnuma, Nami Ishida, Kei Furui Ebisawa, Goh Ohji

**Affiliations:** CarbGeM Inc., Arlington, Massachusetts; CarbGeM Inc., Arlington, Massachusetts; CarbGeM Inc., Arlington, Massachusetts; CarbGeM Inc., Arlington, Massachusetts; CarbGeM Inc., Arlington, Massachusetts; Japan Display Inc., Ebina-shi, Kanagawa, Japan; Japan Display Inc., Ebina-shi, Kanagawa, Japan; Japan Display Inc., Ebina-shi, Kanagawa, Japan; Japan Display Inc., Ebina-shi, Kanagawa, Japan; Japan Display Inc., Ebina-shi, Kanagawa, Japan; Japan Display Inc., Ebina-shi, Kanagawa, Japan; Kobe University Hospital, Kobe, Hyogo, Japan; Kobe University Hospital, Kobe, Hyogo, Japan; Kobe University Hospital, Kobe, Hyogo, Japan; Kobe university hospital, Kobe, Hyogo, Japan

## Abstract

**Background:**

Agar plates are used to culture microorganisms and identify microbes. As the determination is made by a laboratory technician, the detection of bacterial growth on agar plate is highly depends on each microbiologist. Here, we developed a new thin-film transistor (TFT) image sensor for measuring transmitted light and calculating optical density (OD) to observe bacterial growth on agar plates.

**Methods:**

The TFT image sensor consists of 4 image sensor arrays (271 x 436 pixels, pixel size = 80 μm) and flat surface illuminating module. Each pixel measures the intensity of light passing through the agar medium.

Verification of the sensor performance was conducted using Enterobacter cloacae spread on agar medium, which is positioned between the sensor and the illuminating module, in an incubator for 18 hours at 37°C. The intensity of light was measured every 5 minutes from all pixels. For each pixel, the baseline intensity is defined as the average of first 10 frames (I_0_), and OD is calculated from the average intensity of three consecutive frames. Application for inhibition circle measurement was also conducted using Escherichia coli with two antibiotic discs (amoxicillin/clavulanic acid and ampicillin/sulbactam).

**Results:**

Figure 1 shows the OD measured at the pixels in the high concentration area (blue) and single colony area (red) superimposed on the transmitted light image of the last frame. The time needed to detect rising of OD was significantly longer in the low concentration area compared to the high concentration area with exponential curve.

As a direct application of the OD measurement, we attempted to predict bacterial growth. With the threshold set at OD=0.05 and TRUE growth defined as OD >0.1 at the last frame, the precision of the prediction was nearly 100% after 250 min in high concentration area (Figure 2). The decrease in precision after 650 min was due to very slow growth in these areas.

Using the principle above, we successfully predict the inhibition circle diameter at a much earlier time (8.3 hours vs. 25 hours) as shown in Figure 3.

Fig1 OD-Plot

**Figure 1.**
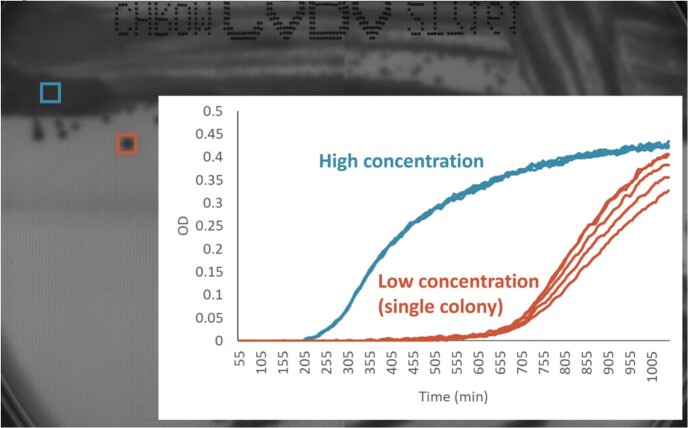
Growth curves measured at the pixels in the high concentration area (blue square) and single colony (red square) superimposed on the transmitted light image of the last frame.

Fig2 prediction

**Figure 2.**
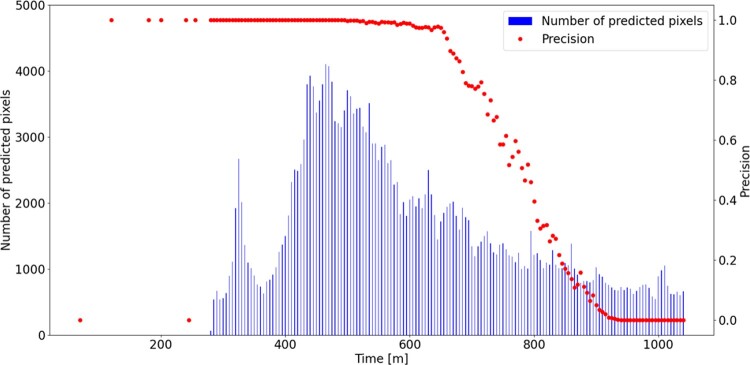
Bacterial growth prediction using optical density.

Fig3 Inhibition circle

**Figure 3.**
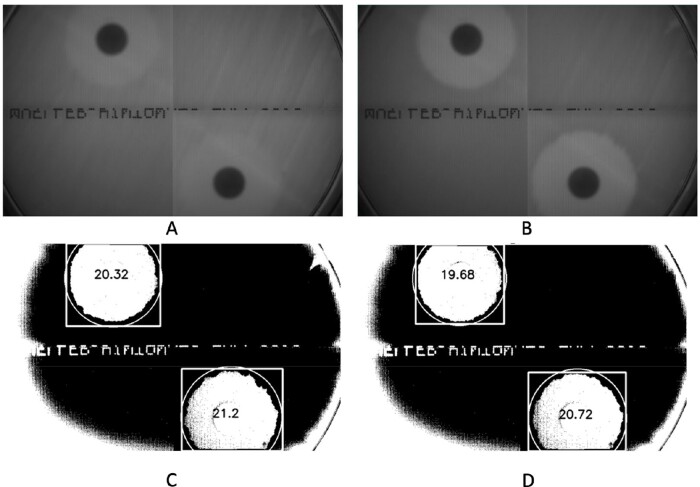
Prediction of the inhibition circle created by two antibiotic discs (top/left: amoxicillin/clavulanic acid and bottom/right: ampicillin/sulbactam) using optical density measurements. Transmitted light image at 8.3 hrs. (A) and 25 hrs. (B). Predicted growth and binarized image from 8.3-hour data with measured diameter of each circle (C). Binarized image from 25-hour data with measured diameters (D).

**Conclusion:**

The new TFT image sensor was able to measure bacterial growth on agar plates. By using the growth curve from each pixel, we were able to predict bacterial growth on agar plates and propose clinical applications such as inhibition circle measurement.

**Disclosures:**

**Masakazu Nakajima, B. Engineering**, Soiken Holdings: Board Member|Welby Inc.: Board Member|Welby Inc.: Ownership Interest|Welby Inc.: Stocks/Bonds **Goh Ohji, MD, PhD**, CarbGeM: Advisor/Consultant|CarbGeM: Grant/Research Support

